# Analysis of Mechanisms for Increased Blood Pressure Variability in Rats Continuously Infused with Angiotensin II

**DOI:** 10.1155/2023/4201342

**Published:** 2023-01-04

**Authors:** Danfeng Jiang, Minami Matsuzaki, Yukiko Kawagoe, Kazuo Kitamura, Toshihiro Tsuruda, Koichi Kaikita, Yujiro Asada, Johji Kato

**Affiliations:** ^1^Frontier Science Research Center, University of Miyazaki Faculty of Medicine, Miyazaki 889-1692, Japan; ^2^Department of Hemo-Vascular Advanced Medicine, University of Miyazaki Faculty of Medicine, Miyazaki 889-1692, Japan; ^3^Department of Internal Medicine, University of Miyazaki Faculty of Medicine, Miyazaki 889-1692, Japan; ^4^Department of Pathology, University of Miyazaki Faculty of Medicine, Miyazaki 889-1692, Japan

## Abstract

**Objective:**

We reported that rats infused with angiotensin II (Ang II) are not only a model of hypertension but also of augmented 24 h blood pressure variability (BPV). In this study, we examined the mechanisms for Ang II-induced BPV, focusing on BP, heart rate (HR), baroreceptor reflex sensitivity (BRS), and medial area of the aortic arch.

**Methods:**

Nine-week-old male Wistar rats were infused with subcutaneous 5.2 *μ*g/kg/h Ang II with or without oral administration with 30 mg/kg/day azelnidipine for 14 days. BP and HR were recorded every 15 min under an unrestrained condition by a radiotelemetry system, while BPV was evaluated by standard deviation of BP. BRS was quantified by a sequence analysis, and medial thickness of the aortic arch was measured by microscopic examination.

**Results:**

BPV increased at days 7 and 14 following continuous infusion of Ang II. Before the infusion, a positive correlation was found between BP and HR, but it became negative at day 7 and then weakened or disappeared at day 14. BRS was slightly impaired at day 7 and significantly lowered at day 14, a phenomenon accompanied by thickened medial area of the aortic arch in Ang II-infused rats. Those Ang II-induced alterations were all significantly attenuated by azelnidipine.

**Conclusions:**

The present findings suggest sequential changes in the mechanisms behind augmented BPV in rats continuously infused with Ang II over 14 days.

## 1. Introduction

Hypertension is an important risk factor for cardiovascular (CV) disease, a major cause of death worldwide. In addition to elevation of blood pressure (BP), increased fluctuation of BP, so called BP variability (BPV), has been recognized as a CV risk factor [1–4]. In clinical settings, there are various types of BPV, from beat-by-beat to visit-by-visit, depending on the duration for which BP levels are evaluated [5, 6]. A number of BP-lowering drugs are currently available in treating patients with hypertension, while little is known about the mechanism or effective drugs for increased BPV. In the past decades, various animal models have been used to study the pathophysiology of hypertension or to develop BP-lowering drugs. Among those animals, rats and mice infused with angiotensin II (Ang II) were often used as a model not only for hypertension but also for cardiovascular organ damage [7–9]. Although few models were available for experimental study of BPV, we recently found that continuous infusion of Ang II widened fluctuation of BP levels over 24 hours in rats, suggesting this animal as a model of hypertension complicated by augmented 24 h BPV [10]. The aim of this study was to explore the mechanisms behind augmentation of BPV in hypertension induced by continuous infusion of Ang II. Accordingly, we examined the relationship between BP and heart rate (HR), alterations in baroreceptor reflex sensitivity (BRS), and medial area of the aortic arch where baroreceptors are located [11], following continuous infusion of Ang II over 14 days. In addition, we treated Ang II-infused rats with azelnidipine to clarify the mechanism of action of Ang II and to examine the effects of calcium channel blocker.

## 2. Materials and Methods

### 2.1. Animals, Chemicals, and BP Monitoring

Eight-week-old male Wistar rats (SLC, Inc., Hamamatsu, Japan) weighing 260 to 300 g were maintained under a 12 h light and 12 h dark cycle in a specific pathogen-free condition with standard chow and water given ad libitum. Ang II peptide was obtained from Peptide Institute, Inc. (Osaka, Japan), and azelnidipine was from Tokyo Chemical Industry Co., Ltd. (Tokyo, Japan). Recording of BP and HR in conscious, unrestrained rats were carried out using a telemetry system (Data Sciences International, St. Paul, MN, USA) with the HD-S10 transmitter implanted in the abdominal cavity, as previously described [10, 12]. In brief, under anesthesia by intraperitoneal injection of 2.0 mg/kg butorphanol tartrate, 1.6 mg/kg midazolam, and 0.12 mg/kg medetomidine hydrochloride, the abdominal cavity was opened by a middle incision, and the aorta was isolated from the retroperitoneal tissue. The catheter inserted into the aorta was secured with tissue adhesive, and the transmitter was then placed in the intraperitoneal space by suturing to the muscular layer of the abdominal wall.

After the 7-day period of recovery from the implantation operation, rats were divided into three groups infused with vehicle or Ang II solution with or without azelnidipine given in rat chow at 30 mg/kg/day over 14 days. Ang II dissolved in 0.1 mol/l acetic acid was subcutaneously infused into rats at a rate of 5.2 *μ*g/kg/h over 14 days via an osmotic minipump (Alzet Model 2002; Durect, Cupertino, CA, USA). The number of rats examined was 8 for each group. We carried out 24 h BP monitoring before and after 7 and 14 days of the infusion, where BP and HR were measured every 15 min (48-point measurements over 12 h). Systolic and diastolic BP (SBP and DBP) values for each animal were obtained by averaging 48 measurements during 12 h of the light or dark cycle. Meanwhile, BPV was evaluated using the standard deviation (SD) of those SBP and DBP values of 48-point measurements during 12 h of the light or dark cycle. The present study was performed in accordance with the Animal Welfare Act and with approval of the University of Miyazaki Institutional Animal Care and Use Committee (No. 2017-509-4). This manuscript was prepared according to the ARRIVE guidelines.

### 2.2. Microscopic Examination

After the BP recording, the animals were sacrificed by decapitation; then, the whole aorta was immediately excised and fixed in 10% formaldehyde. The aortic arch just distal to the left subclavian artery was embedded and sectioned at 2 *μ*m thickness. The aortic sections stained with hematoxylin-eosin were quantitatively evaluated by a single observer in a blind manner. Cross-sectional areas of the sections were determined by computerized measurement with a WinROOF2018 software (MITANI Corp., Tokyo, Japan), in which medial area was obtained by subtracting the area surrounded by the internal elastic lamina from that surrounded by the external elastic lamina (Supplemental Figure 1).

### 2.3. Calculation of BRS and Statistical Analysis

To estimate BRS, data obtained from continuous BP monitoring were analyzed by a sequence technique with the software HemoLab before 7 and 14 days of the experiment period, as reported previously [13, 14]. This method identified spontaneous sequences of four or more heart beats, where BP levels and pulse intervals changing in the same direction were linearly correlated at an *r* value greater than 0.8, and the mean of slopes of regression lines was used as an index of BRS. Averages of the mean BRS values calculated from the continuous BP monitoring data during the light cycle of 10 : 00 to 14 : 00 and the dark cycle of 22 : 00 to 2 : 00 were obtained in the present study.

All data were analyzed statistically with IBM SPSS software version 26.0 (IBM, Armonk, NY, USA). Multiple comparisons were made with one-way analysis of variance and the Tukey-Kramer method. Simple regression analysis was used to examine the relationship between two variables. All data are expressed as the mean ± S.E., and *P* < 0.05 was considered to be significant.

## 3. Results

**Figure 1:**
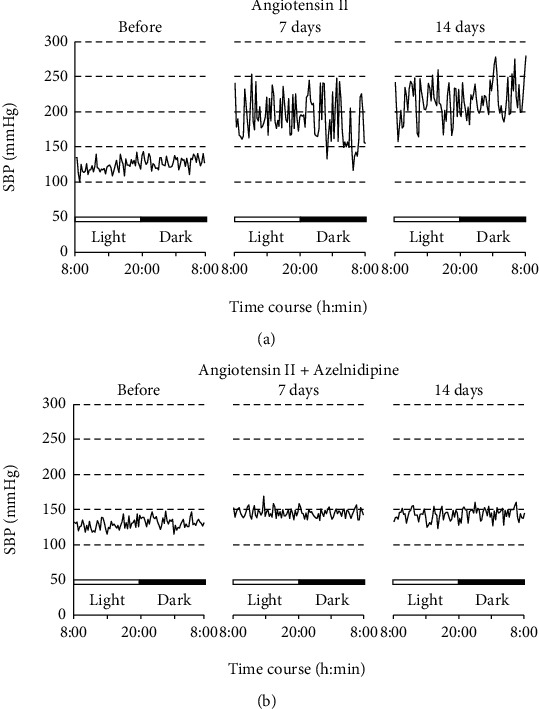
Representative 24 h records of SBP of rats subcutaneously infused with angiotensin II with (b) or without (a) oral administration of azelnidipine over 14 days. SBP levels were measured every 15 min by a telemetry system for 24 hours before and after 7 and 14 days of the infusion.

Figures 1(a) and (b) are representative 24 h records of SBP of rats subcutaneously infused with Ang II with or without azelnidipine, respectively. Ang II not only elevated BP levels but also augmented variabilities of those recorded every 15 min at day 7 and 14, while the long-acting CCB azelnidipine attenuated BP elevation and augmentation of the variability. As shown in Supplemental Figures 2 to 4, results consistent with our previous reports were obtained in averages and SDs of SBP, DBP, and HR; variabilities of SBP and DBP were augmented by Ang II infusion at days 7 and 14, while those of HR increased at day 7 and then declined at day 14 [10, 15]. Meanwhile, treatment with azelnidipine mostly alleviated those augmentations of BP and HR. [15].

**Figure 2:**
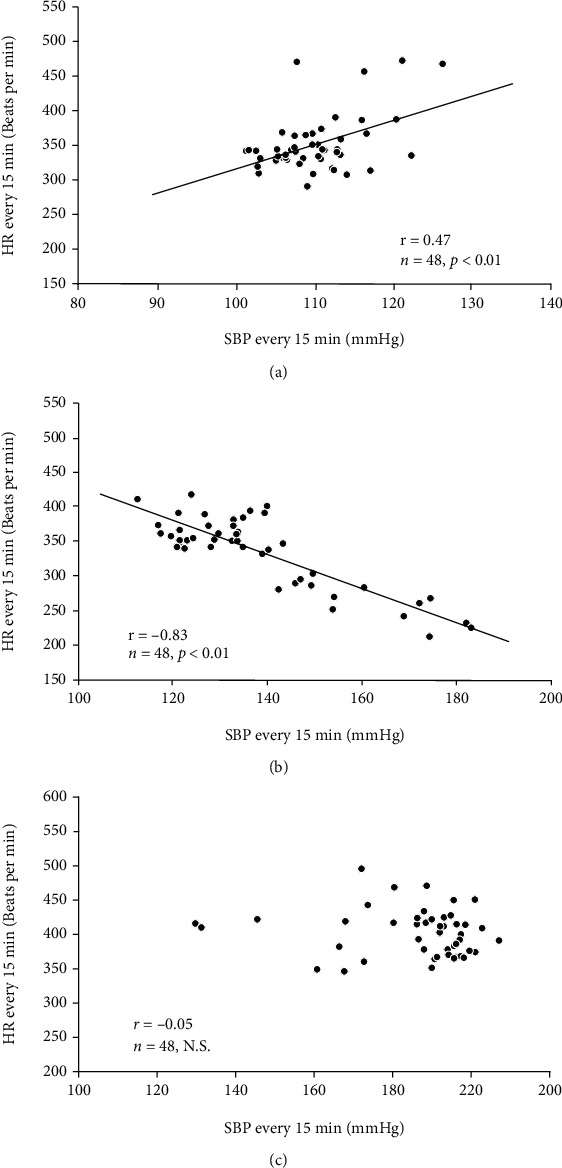
Representative plots for relationships between systolic blood pressure (SBP) and heart rate (HR) monitored every 15 min during the light cycle of 12 hours before (a), 7 days (b), and 14 days (c) following continuous infusion of angiotensin II. N.S.; not significant.

**Figure 3:**
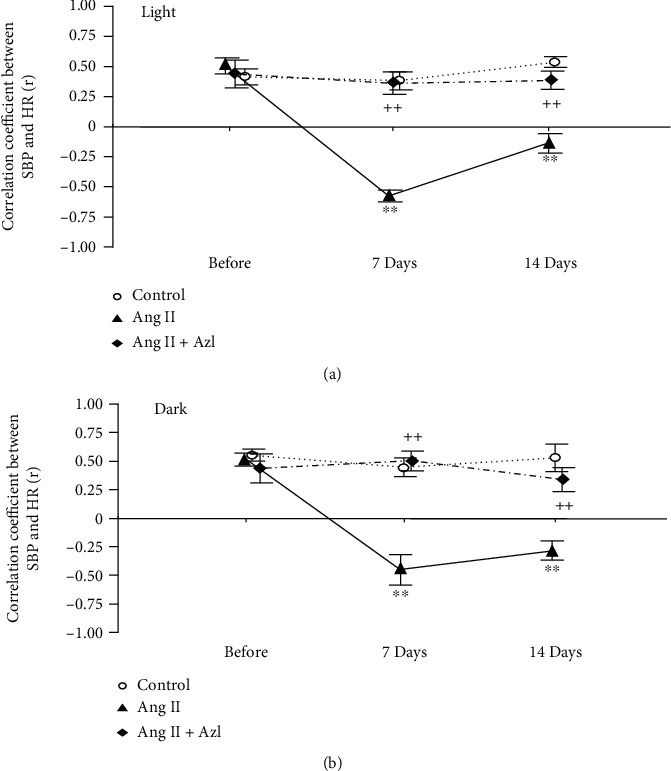
Time course of correlation coefficient (*r*) for the relationships between systolic blood pressure (SBP) and heart rate (HR) during the light (a) and dark (b) cycles of 12 hours in control rats and those infused with angiotensin II (Ang II) with or without oral administration of azelnidipine (Azl). Mean ± S.E.; *n* = 8; ^∗∗^*P* < 0.01, vs. control; ^++^*P* < 0.01, vs. Ang II alone.

We analyzed the relationships between BP and HR monitored every 15 min by a simple regression analysis before and after 7 and 14 days of Ang II infusion.  Figure 2 shows representative plots of SBP and HR values of a rat infused with Ang II during the light cycle. Before the infusion, a significant positive correlation was observed between SBP and HR, but interestingly, this correlation became negative or inverse at day 7 and then insignificant at day 14. Figures 3(a) and 3(b) are time courses of averages of correlation coefficients (*r*) calculated by a simple regression analysis of the relationships between SBP and HR in three groups. As shown, *r* values of the control group remained unchanged during the experimental period, while we found notable changes in the Ang II group; *r* became negative at day 7 of the infusion and then came close to zero at day 14. Meanwhile, treatment with azelnidipine mostly inhibited these changes in *r* values of Ang II-infused rats.

**Figure 4:**
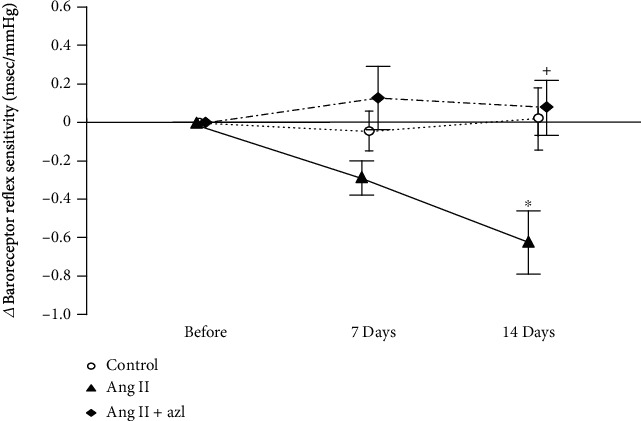
Changes in baroreceptor reflex sensitivities (BRSs) following the infusion of angiotensin II (Ang II) with or without oral administration of azelnidipine (Azl) over 14 days. Data shown are the differences between BRSs before and 7 or 14 days of the infusion (*Δ*BRSs). Mean ± S.E.; *n* = 8; ^∗^*P* < 0.05, vs. control; ^+^*P* < 0.05, vs. Ang II alone.

Next, we quantified BRS using a sequence technique in order to examine the mechanism behind the sequential change in the relationship between BP and HR and the augmentation of BPV following Ang II infusion. The basal values for calculated BRSs before the infusion of Ang II were as follows: control, 1.83 ± 0.11; Ang II, 2.07 ± 0.10; Ang II plus azelnidipine, 1.42 ± 0.13 msec/mmHg.  Figure 4 shows changes in BRSs (*Δ*BRS) following Ang II infusion with or without azelnidipine over 7 and 14 days. Ang II Infusion slightly reduced BRS at day 7, and significant reduction was seen at day 14, while azelnidipine alleviated this reduction.

**Figure 5:**
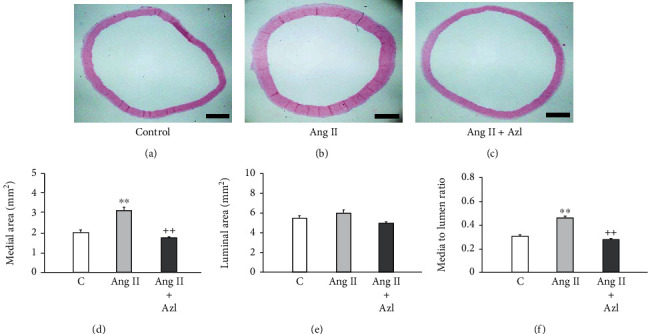
Morphological analyses of sections of the aortic arch from control rats and those infused with angiotensin II (Ang II) with or without oral administration of azelnidipine (Azl) over 14 days. (a–c) Representative photos of hematoxylin eosin-stained aortic sections. (d–f) Quantitative analyses of medial and luminal area and media to lumen ratio. Mean ± S.E.; *n* = 8; ^∗∗^*P* < 0.01, vs. control; ^++^*P* < 0.01, vs. Ang II alone; bars, 1.0 mm.

We examined rat aortic sections to see whether the above-mentioned alterations of BRS were related to any morphological change in the aortic arch, where baroreceptors are located [11]. Figures 5(a)–5(c) are representative photos of hematoxylin-eosin-stained sections of the aortic arch at 12.5x magnification. Compared with the control, aortic media of a rat infused with Ang II was thicker; azelnidipine suppressed the medial thickening induced by Ang II. Figures 5(d)–5(f) show results of statistical analyses for cross-sectional area of the aortic arch. Ang II increased cross-sectional area of the medial layer, but this increase was attenuated by azelnidipine (Figure 5(d)). As shown in Figure 5(e), no significant difference was noted in the luminal area among three groups; therefore, similar results were obtained in media to lumen ratio as in medial area (Figure 5(f)). To look at the relationships between changes in BPV, BRS, and medial area, we examined data at day 14 from all the study groups by a simple regression analysis. Changes in SD of SBP inversely correlated with those in BRS, which were further associated with medial area of the aortic arch (Supplemental Figure 5).

Shown in Supplemental table are the heart weight to body weight (HW/BW) ratio and locomotive activity of three groups at the end of the experiment. The HW/BW ratios were increased following infusions of Ang II. Locomotive activities of three groups at the dark cycle were higher than those at the light, but no differences were noted between the study groups at both cycles.

## 4. Discussion

Consistent with our previous reports [10, 15], augmented 24 h BPVs were similarly seen at days 7 and 14 in Ang II-infused rats in the present study, while we found sequential changes in the relationships between BP and HR recorded every 15 min. Before Ang II infusion, a significant positive correlation between BP and HR was observed during the light and dark cycles, suggesting that the physiological BP fluctuation every 15 min depends on sympathetic and/or parasympathetic activity. However, this relationship between BP and HR became totally opposite or negative at day 7 of the infusion, and thereafter, it became much weaker or disappeared at day 14.

To interpret these sequential changes in the relationship between BP and HR at days 7 and 14, we need to discuss the mechanisms for BP elevation following continuous infusion of Ang II. Although the precise mode of action remains to be specified, basically two mechanisms have been considered for hypertension induced by Ang II infusion [16, 17]. One is increased peripheral vascular resistance caused by constriction and/or remodeling of the arterioles, and the other is renal sodium retention that may result in increase of cardiac output [16, 17]. Given the widely fluctuating BP levels every 15 min at day 7, we consider fluctuating magnitude of vasoconstriction of the resistance arteries by Ang II infusion, while alteration in renal sodium retention unlikely occurs in such a short period of time. In the present study, we obtained BP value every 15 min by averaging BP levels monitored continuously for 10 sec. Therefore, we speculate that such fluctuation in peripheral resistance might have occurred in a time period longer than 10 sec but shorter than 15 min in the Ang II-infused group. In addition, the negative correlation between BP and HR at day 7 could be explained by baroreflex; when BP goes up, HR goes down because of suppressed sympathetic and/or increased parasympathetic outflow via baroreflex loop and vice versa. Accordingly, as shown in Supplemental Figure 4, HR variability could also have been augmented at day 7 by wide fluctuation of BP levels. Because a major pharmacological action of CCB is an inhibition of vasoconstriction of resistance arteries [18], the suppressive effect of azelnidipine on BPV observed in this study is consistent with the hypothesis of fluctuation in peripheral resistance. Thus, BPV augmented by Ang II infusion seems largely dependent on this fluctuation at day 7.

Next, we discuss the phenomena observed at day 14 of Ang II infusion in this study. Augmented BPV at this time point was accompanied by three phenomena: one was that the relationship between BP and HR weakened or mostly disappeared, a finding in contrast to those before and after 7 days of Ang II infusion. The other two findings were significant impairment of BRS and thickening of medial area of the aortic arch in the Ang II-infused group. Impaired BRS is accordant with the weaken relationship between BP and HR and with the fact that augmented HR variability at day 7 diminished or returned to the basal level at day 14. This may be because impaired baroreceptor reflex was unable to sufficiently alter sympathetic and/or parasympathetic outflow in response to fluctuation of BP.

To discuss the mechanism for impaired BRS by Ang II, the only data we have is measurement of medial area of the aortic arch. According to previous clinical studies, augmented BPVs were closely associated with stiffened large arteries in human patients [5, 19, 20]. Therefore, we speculate that stiffened aortic arch by medial thickening impaired baroreceptor function, and indeed, pulse pressure, a possible marker for stiffened large arteries [21], was found to increase following Ang II infusion (Supplemental Figure 6). Consistent with those assumptions, a significant correlation was noted between the medial area and impaired BRS (Supplemental Figure 5); however, we have no direct, quantitative data about stiffness of the aortic wall, so this hypothesis needs to be verified in future experiments.

In this study, the long-acting CCB azelnidipine suppressed BPV augmented by Ang II infusion at days 7 and 14. Fluctuating vascular resistance might have also been a mechanism for augmented BPV at day 14; meanwhile, we need to raise a possibility that impaired BRS is involved in augmented BPV at day 14. Indeed, azelnidipine inhibited the variability, alleviating impairment of BRS and thickening of aortic media, though the detailed mechanism for those actions remains to be specified. Nonetheless, the present findings are comparable with the clinical notion that long-acting CCBs effectively suppress BPV in comparison with other classes of antihypertensive drugs [22–24]. When discussing the effects of azelnidipine observed this study, it is also an important question whether the alterations found in the Ang II-infused group without azelnidipine were attributed to BP elevation per se. We assume that these Ang II-induced alterations are at least partly independent of BP elevation. This is based on our previous report where we found that treatment with hydralazine attenuated BP elevation but not BPV augmentation in rats infused with Ang II [15].

## 5. Conclusion

In summary, we explored the mechanisms behind increased 24 h BPV of rats continuously infused with Ang II for 14 days. Augmented BPV at day 7 of the infusion was accompanied by an inverted relationship between BP and HR, but this correlation was weakened or disappeared at day 14. Those changes were associated with progressive impairment of BRS over 14 days, and impaired BRS at day 14 was accompanied by thickened medial layer of the aortic arch. All the sequential changes were attenuated by treatment with azelnidipine.

## Data Availability

We have no underlying data related to this article.

## Supplementary Materials

Supplementary Materials Please find the supplemental material available for this article. All supplemental material carries the same license as the article it is here associated with.
