# Supplementary material for: Analysis of Mechanisms for Increased Blood Pressure Variability in Rats Continuously Infused with Angiotensin II
Source: J Renin Angiotensin Aldosterone Syst. 2023 Jan 4;2023:4201342. doi: 10.1155/2023/4201342 (PMC9833913; doi:10.1155/2023/4201342)
Supplement: Supplementary Materials — Please find the supplemental material available for this article. All supplemental material carries the same license as the article it is here associated with. [file 4201342.f1.pdf]

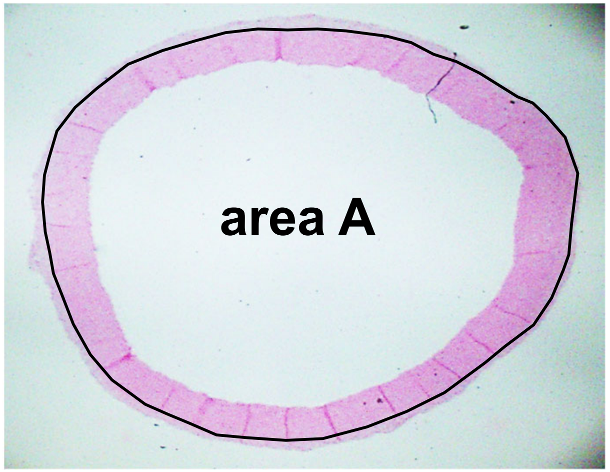

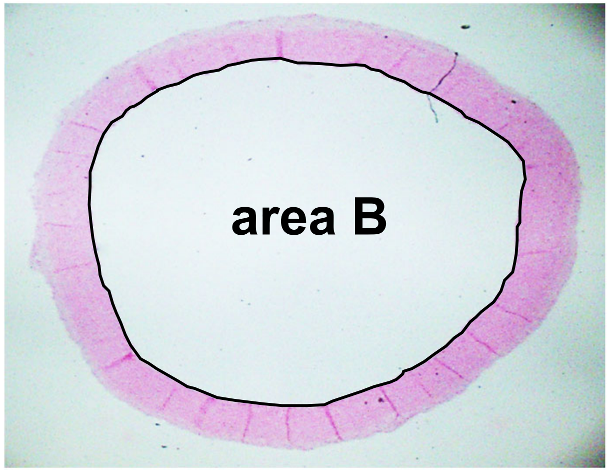

Supplemental figure 1. Measurement of the medial area of rat aorta. Medial area was calculated by subtracting the area surrounded by the internal elastic lamina (**area B**) from that surrounded by the external elastic lamina (**area A**): medial area = **area A** - **area B**.

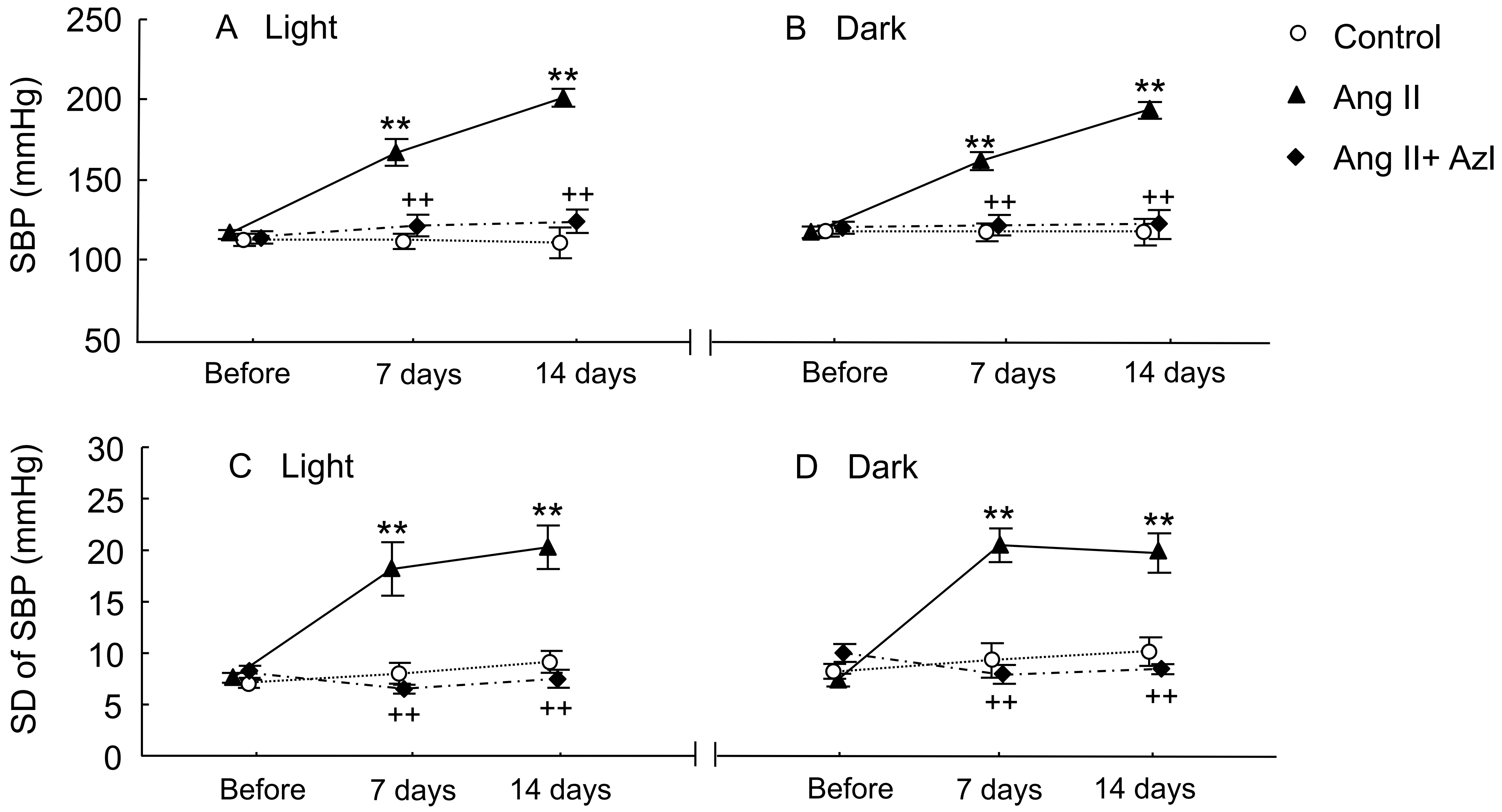

Supplemental figure 2. SBP and standard deviation (SD) of SBP during the light (A and C) and dark (B and D) cycles of 12 hours in control rats and those infused with angiotensin II (Ang II) with or without oral administration of azelnidipine (Azl). Mean ± S.E.; n=8; **P<0.01, vs. control; ++P<0.01, vs. Ang II alone.

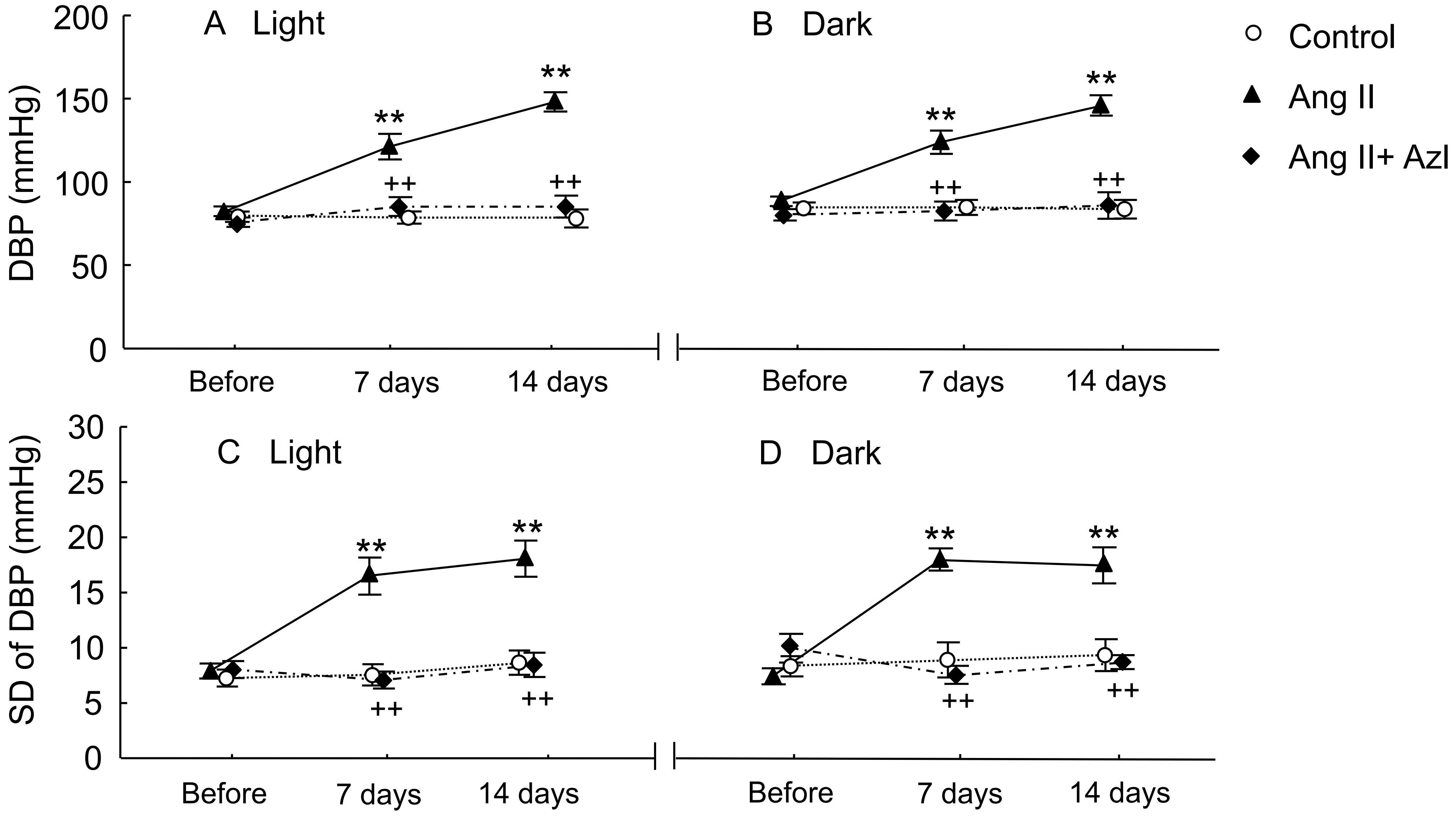

Supplemental figure 3. DBP and standard deviation (SD) of DBP during the light (A and C) and dark (B and D) cycles of 12 hours in control rats and those infused with angiotensin II (Ang II) with or without oral administration of azelnidipine (Azl). Mean ± S.E.; n=8; **P<0.01, vs. control; ++P<0.01, vs. Ang II alone.

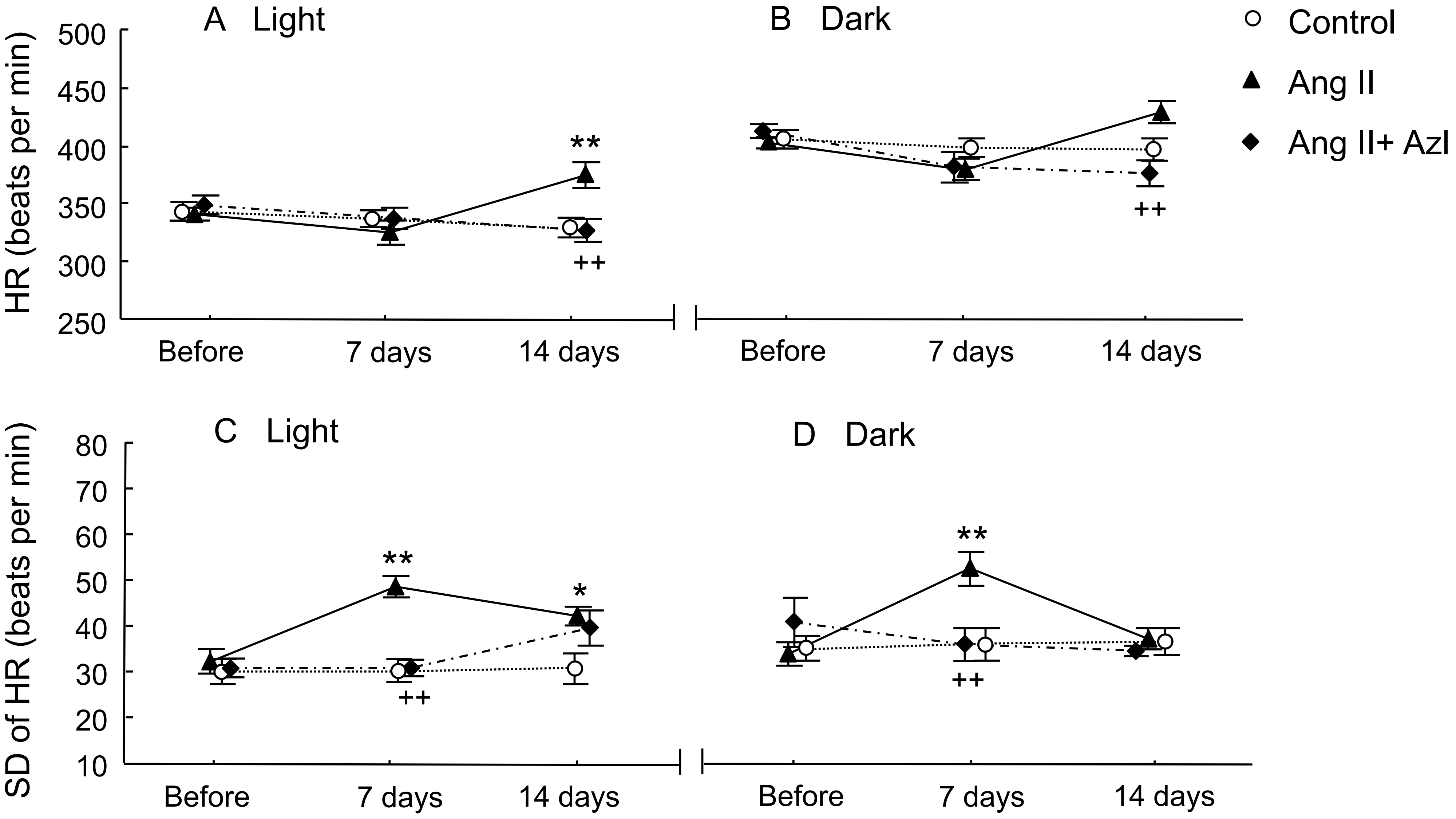

Supplemental figure 4. Heart rate (HR) and standard deviation (SD) of HR during the light (A and C) and dark (B and D) cycles of 12 hours in control rats and those infused with angiotensin II (Ang II) with or without oral administration of azelnidipine (Azl). Mean ± S.E.; n=8; *P<0.05, **P<0.01, vs. control; ++P<0.01, vs. Ang II alone.

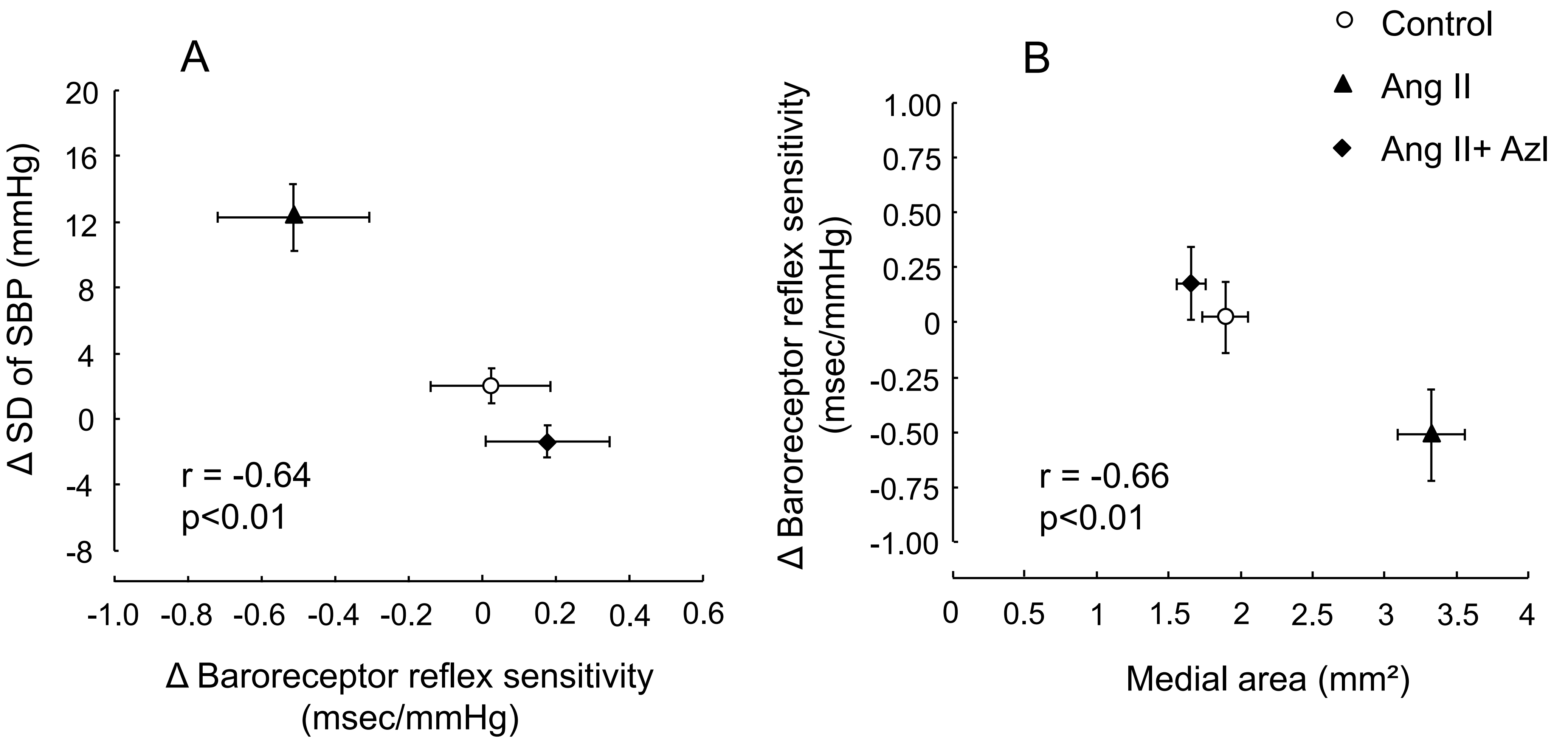

Supplemental figure 5. Relationships between changes in standard deviation of SBP (ΔSD of SBP) and baroreceptor reflex sensitivity (ΔBRS) (**A**) and between ΔBRS and medial area (**B**) following angiotensin II (Ang II) infusion with or without oral administration of azelnidipine (Azl) over 14 days. Correlation coefficients (r) of the relationships between two variables were obtained by a simple regression analysis using data from all the rats of three study groups at day 14 (n=24). The means ± S.E. of each study group are shown in panels A and B.

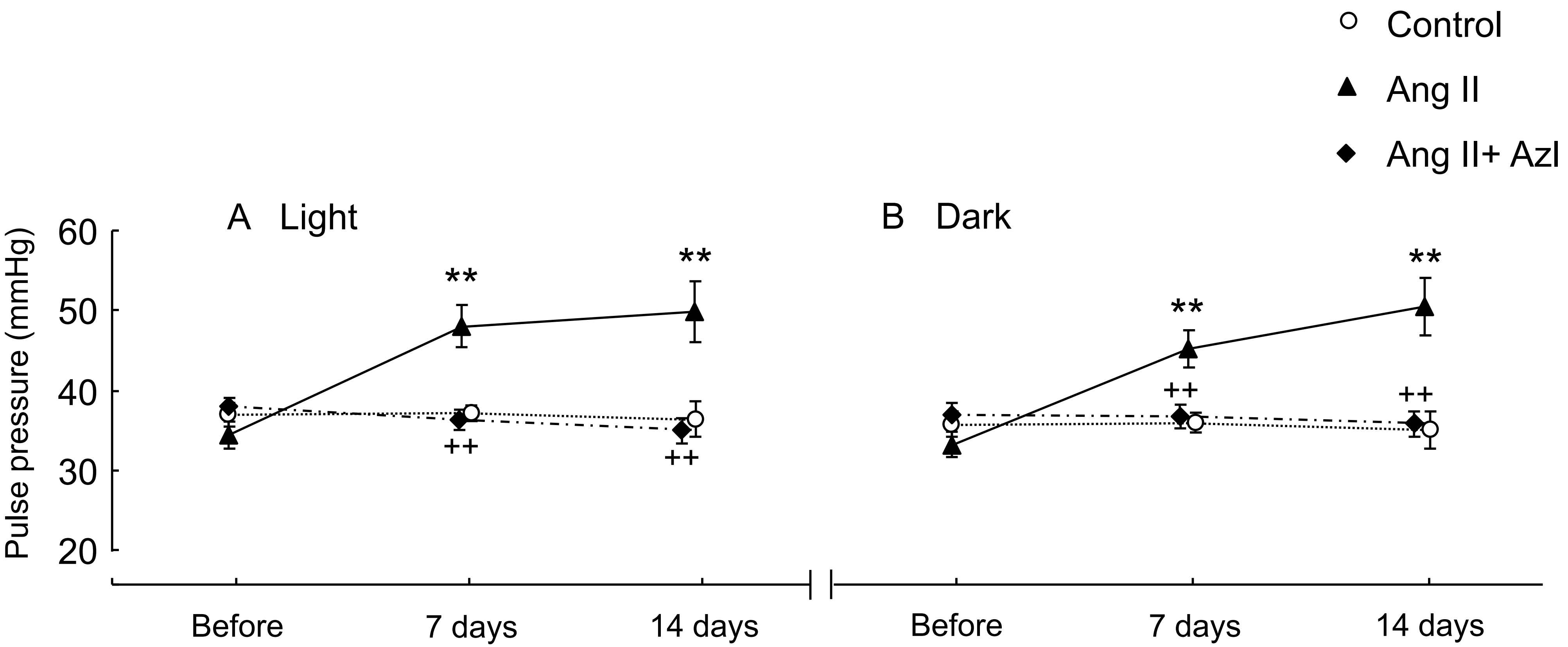

Supplemental figure 6. Pulse pressure (PP) during the light (A) and dark (B) cycles of 12 hours in control rats and those infused with angiotensin II (Ang II) with or without oral administration of azelnidipine (Azl). Mean ± S.E.; n=8; **P<0.01, vs. control; ++P<0.01, vs. Ang II alone.

Supplemental table. Heart weight (HW) to body weight (BW) ratio and locomotive activity of control and Ang II-infused rats with or without Azl for 14 days

	Control	Ang II	Ang II + Azl
HW/BW ratio ($\times 10^{-3}$)	2.53 ± 0.03	3.44 ± 0.12**	3.19 ± 0.05$^{*,+}$
Activity Light (unit)	1.53 ± 0.12	1.56 ± 0.21	1.06 ± 0.09
Dark (unit)	3.87 ± 0.26	3.96 ± 0.44	3.17 ± 0.25

Ang II, angiotensin II; Azl, azelnidipine.
Mean ± S.E; *$P < 0.05$, **$P < 0.01$, vs. control; $^{+}P < 0.05$, vs. Ang II alone.
